# Role of Phytohormones in Biomass and Polyphenol Accumulation in *Salvia bulleyana* In Vitro Culture

**DOI:** 10.3390/biom13020227

**Published:** 2023-01-24

**Authors:** Izabela Grzegorczyk-Karolak, Marta Krzemińska, Anna K. Kiss, Aleksandra Owczarek-Januszkiewicz, Monika A. Olszewska

**Affiliations:** 1 Department of Biology and Pharmaceutical Botany, Medical University of Lodz, Muszynskiego 1, 90-151 Lodz, Poland; 2 Department of Pharmacognosy and Molecular Basis of Phytotherapy, Warsaw Medical University, Banacha 1, Warsaw 02-097, Poland; 3 Department of Pharmacognosy, Medical University of Lodz, Muszynskiego 1, 90-151 Lodz, Poland

**Keywords:** benzylaminopurine riboside, kinetin, N-benzylotetrahydropyranyl adenine, meta-topoline, phytohormones, rosmarinic acid, TOPSIS analysis

## Abstract

*Salvia bulleyana* is a plant native to the Chinese Yunnan Province. This species has been used in traditional Chinese medicine as a substitute for Danshen (the roots of *Salvia miltiorrhiza*). The aim of our study was to establish an effective system for propagating *S. bulleyana* shoots to obtain large amounts of material rich in bioactive compounds. Phytohormones were used to regulate shoot growth and regeneration potential and influence plant secondary metabolism. The shoot tips were incubated on a Murashige and Skoog agar medium supplemented with 0.1 or 0.5 mg/L IAA (indole-3-acetic acid) and the cytokinins benzylaminopurine (BAP), meta-topoline (M-T), 6-benzylaminopurine riboside (RBAP), N-benzyl-9-(2-tetrahydropyranyl)-adenine (BPA) or kinetin, (K) at concentrations of 0.5, 1 or 2 mg/L. It was observed that the type and concentration of growth regulator significantly influenced the regeneration potential of *S. bulleyana* shoots. The highest multiplication rate was obtained when 0.1 mg/L IAA and 2 mg/L BPA were used. Under these conditions, 100% of shoot tips formed buds and almost seven buds/shoot per explant were obtained after five weeks. Meanwhile, the highest biomass was found for shoots growing on a medium supplemented with 0.1 mg/L IAA and 1 mg/L M-T: 1.2 g of fresh weight and 0.17 g of dry weight. However, a medium with 0.1 mg/L IAA and 2 mg/L RBAP was most favorable for bioactive phenolic acid content, with a total polyphenol level (37.7 mg/g dw) 4.5 times higher than in shoots grown on medium without growth regulators (8.23 mg/g dw). Finally, optimal conditions were selected by TOPSIS (technique for order of preference by similarity to the ideal solution); the culture of *S. bulleyana* grown on an MS medium containing 0.1 mg/L IAA and 1 mg/L M-T was found to be the most efficient for polyphenol accumulation and can be used for the production of medicinally relevant compounds.

## 1. Introduction

In vitro plant cultures represent a potential efficient source of bioactive secondary metabolites. Such cultures demonstrate a short initiation time, independence from seasonality and climatic conditions and the possibility of producing new compounds—normally not found in the source plant [1,2]. However, the success of a plant culture depends on many factors, including culture genotype, growth conditions and the components of the culture medium. Among the latter, plant hormones play a particularly important role [3].

Phytohormones are biodegradable low-molecular-weight chemical messengers that regulate plant development and metabolism [3,4]. The exogenous application of growth regulators into the growth medium influences the growth of in vitro cultures and might promote a crop yield, with the results depending on both the type and concentration of phytohormone [5]. In plant biotechnology, natural and synthetic auxins and cytokinins are mainly used. Auxins are involved in cell division, cell enlargement and organ development, whereas cytokinins take part in many basic processes, including photosynthesis, maintenance of cell proliferation, cell differentiation and retardation of senescence. BAP has been the most widely used cytokinin in plant biotechnology for several decades. However, recently, there have been reports of media supplementation with various BAP derivatives, such as RBAP, BPA or M-T. These reports, although scarce, seem promising, both in terms of growth and bioactive metabolite accumulation [6,7].

Sage species have been used in the medicine of all cultures for centuries. The roots and shoots of *Salvia* plants contain a remarkably broad spectrum of secondary metabolites responsible for a wide range of pharmacological activities, demonstrating antioxidant, anti-inflammatory, antitumor, antiviral and antibacterial properties [8,9]. In Chinese medicine, *Salvia miltiorrhiza* is the best-known species, but around 40 other sages are locally used as substitutes, including *S. bulleyana* [10]. They have been used for treating cardiovascular, immune, hepatic and renal system diseases, and insomnia.

*S. bulleyana* (Zi Danshen) is a perennial plant that grows in the Chinese Yunnan Province. The main group of metabolites identified in its aerial and underground parts are hydrophilic phenolic acids, so these are most likely the secondary metabolites responsible for the biological activity of the raw material [11,12]. However, an obstacle to wider use and knowledge of the properties of *S. bulleyana* is the limited availability of this only locally occurring species. A previous biotechnological study on *S. bulleyana* examined the organogenic competence of various phytohormone combinations, to develop an efficient propagation strategy via leaf explants [13]. The shoots were regenerated in the presence of various cytokinins and then transferred onto an MS medium with BAP, the most commonly described cytokinin used for shoot proliferation of various *Salvia* species [14,15,16]. However, BAP does not yield spectacular results in the propagation of most *Salvia* spp., which prompts the search for other alternatives.

Biotechnological methods can be used not only to obtain a significant amount of plant material that is rarely found in the natural environment but can also significantly increase the level of bioactive compounds by modifying the cultivation conditions [3,5,17]. The aim of the study was to create an efficient system for propagating *S. bulleyana* that could be used to obtain a phenol-rich stock of plant material. The present study investigates the influence of different combinations of plant growth regulators on the growth and accumulation of phenolic acids in shoot culture. In the study, IAA as auxin and five cytokinins (K, BAP, BPA, RBAP and M-T) were used. The investigated cytokinins were provided in three concentrations (0.5, 1 and 2 mg/L) and auxin in two concentrations (0.1 and 0.5 mg/L). The presented work is the first such comprehensive report on the phytohormone-regulation of polyphenol metabolism in sage; it tests the effect of six phytohormones in 32 combinations on culture growth and proliferation and the accumulation of 15 bioactive constituents.

## 2. Materials and Methods

### 2.1. Plant Material

In vitro cultivated shoots of *Salvia bulleyana*, initiated from sterile germinated seeds, were used as the initial material. The seeds were obtained from the Botanical Garden of the University of Bonn (Bonn, Germany) (50°43′28.9″N 7°05′34.6″E). The procedure of seed sterilization, their germination and primary culture development were described earlier [13]. Voucher specimens of the shoot culture were deposited in the Department of Biology and Pharmaceutical Botany, Medical University of Lodz (voucher no. IG/SBS1-1/2019). The culture was grown on Murashige and Skoog (MS) basal medium [18] with 0.1 mg/L IAA and 0.5 mg/L BAP. Fragments of shoots with a tip bud and a single node about 1 cm long (0.056 ± 0.005 g dry weight) were placed on an MS medium supplemented with 0.1 or 0.5 mg/L auxin (IAA) and various cytokinins (BAP, M-T, RBAP, BPA, K) at one of three concentrations: 0.5, 1 or 2 mg/L. 

### 2.2. Culture Conditions

All the culture media were supplemented with 3% sucrose and 0.7% agar. The pH of the media was adjusted to 5.7–5.8. The shoots were grown in 150×4 mm glass tubes containing 25 mL of medium. Media were autoclaved for 19 min at a pressure of 1.1 kg/cm^2^ and 121 °C. The cultures were incubated in a controlled growth chamber for five weeks at 24 ± 2 °C and with a photoperiod of 16 h. The light was provided by fluorescent tubes of cool white light, which provided a light intensity of 40 µm/m^2^∙s.

The growth potential of the culture was measured by estimating the percentage of explants that formed buds, the number of regenerated buds and shoots produced per explant (proliferation coefficient), the length of the main shoots and the dry weight (dw) of the culture on each of the media after a five-week incubation.

### 2.3. Phytochemical Analysis

#### 2.3.1. Sample Preparation

The dry lyophilized plant material for each treatment was ground using a grinder, and ground samples (0.1 g) were extracted with 30 mL of 80% methanol in a sonication bath (Techpan, Warsaw, Poland) at 40 °C for 15 min. The extractions were repeated twice more with less solvent (10 mL). The filtered extracts were combined, evaporated to dryness and stored in a refrigerator (4 °C) before analysis.

#### 2.3.2. Phenolic Acid Qualitative and Quantitative Analysis

The phenolic profile of the hydromethanolic extract of the *S. bulleyana* shoots was determined by UPLC-DAD/ESI-MS/MS using a UPLC-3000 RS system (Dionex, Germany) equipped with an AmaZon SL ion trap mass spectrometer according to Grzegorczyk-Karolak et al. [12]. Compounds were analyzed in a negative ion mode and identified by comparing the information of their retention time, UV–vis and mass spectra with literature data [12,19,20].

For the quantitative HPLC-DAD assay, the samples were chromatographed using an Elite LaChrom Hitachi system (Merck, Darmstadt, Germany) according to Wojciechowska et al. [19]. Peaks were identified on the basis of their retention time and by comparing spectra with those of isolated compounds in a standard solution or literature data [12,19,20]. For quantification, calibration curves were generated by plotting the peak area of the standard compounds at each level versus the concentration of the sample. Quantitative determination was performed using the following reference standards: caffeic acid purchased from Sigma Aldrich/Merck (Darmstadt, Germany), rosmarinic acid (RA) from Extrasynthese (Genay, France) and salvianolic acid A and salvianolic acid F from ChemFaces (Hubei, China). When an authentic standard was not available, the phenolic compounds were quantified according to the calibration curve of an appropriate similar standard: caffeoyl-threonic acid isomers as caffeic acid; methyl rosmarinate, RA hexoside, protolithospermic acid isomers, caffeic acid derivative and dehydrorosmarinic acid as RA; salvianolic acid K and a lithospermic acid isomer as salvianolic acid A; and isomers of salvianolic acid F as salvianolic acid F. Two minor analytes (dehydrorosmarinic acid and a caffeic acid derivative) were quantified as a sum, due to poor separation and low contents. The concentrations of the compounds were calculated as mg/g dry weight (dw) by comparing the peak areas of the compounds to those of the standards. Total phenolic content was calculated as the sum of the content of all quantified phenolic acids.

### 2.4. Establishment of Optimal Growth Conditions

The optimal phytohormone combination for growth was chosen from a given set of alternatives using the technique for order preference by similarity to the ideal solution (TOPSIS). This method assumes that the best alternative is both closer to the positive ideal solution and far away from the negative ideal solution; it has already been successfully used to optimize the composition of growth media (vitamin and sugar content) for the cultivation of hairy roots of *S. bulleyana* [21].

The method consists of selecting decision parameters, normalizing them and assigning appropriate weights to them. Following this, the ideal best (V+) and ideal worst (V-) values are determined for each parameter, and the distance from the ideal best and ideal worst is defined for each alternative. Finally, the performance score is calculated for each treatment, and the one with the highest score is considered optimal. Four decision parameters of culture productivity were selected: dry weight of culture, multiplication ratio, RA content and total phenol content. Detailed calculations were made on the basis of the formulas described earlier [21].

### 2.5. Statistical Analysis

All experiments were repeated three times with 10 explants per treatment (passages 24–26). The data were subjected to analysis of variance (ANOVA) using Statistica 13.1 PL for Windows (StatSoft Inc., Krakow, Poland). Significantly different means were separated using Tukey’s post hoc test (*p* < 0.05). The TOPSIS calculations were performed using Microsoft Excel 2019 software.

## 3. Results and Discussion

### 3.1. Effect of Phytohormones on Culture Growth

To determine the optimal type and concentration of cytokinins and auxin for the multiplication of *Salvia bulleyana* shoots, the apical parts of the shoots were placed on solid MS mediums with auxin IAA (0.1 and 0.5 mg/L) and one of the cytokinins: BAP, RBAP, M-T, BPA and K (0.5, 1 and 2 mg/L). Shoots were grown for five weeks. 

It was previously reported that the addition of a low concentration of auxin to a cytokinin had a positive effect on shoot regeneration and growth [22]. The auxin of choice for the study was IAA. This auxin has been extensively studied and often used in various in vitro plant cultures, including *Salvia* spp. [14,22,23]. The two IAA concentrations used in the present tests (0.1 mg/L and 0.5 mg/L) were selected on the basis of their beneficial effects on growth and proliferation of other sage species shoots [14,22,23]. The inclusion of a strong, synthetic auxin in the medium or the use of a higher concentration could promote callus formation instead of organogenesis [14].

**Figure 1:**
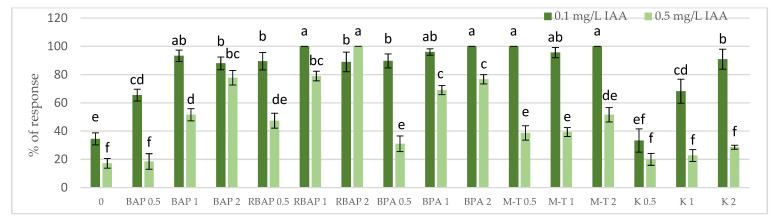
Effect of cytokinin type (BAP, RBAP, BPA, M-T and K) and concentration (0.5, 1 and 2 mg/L) on the proliferative response (%) of *S. bulleyana* culture. Control (0)—shoots cultivated on MS medium only with auxin (IAA—0.1 mg/L and 0.5 mg/L). The values represent the mean ± standard error of three independent experiments. Means marked with the same letter were not significantly different, according to the ANOVA test followed by the post hoc Tukey’s test for multiple comparison (*p* < 0.05).

**Figure 2:**
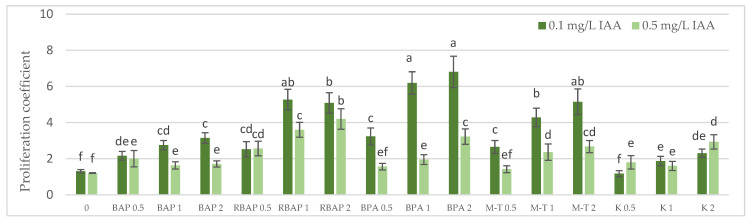
Effect of cytokinin type (BAP, RBAP, BPA, M-T and K) and concentration (0.5, 1 and 2 mg/L) on the proliferative coefficient of *S. bulleyana* culture. Control (0)—shoots cultivated on MS medium only with auxin (IAA—0.1 mg/L and 0.5 mg/L). The values represent the mean ± standard error of three independent experiments. Means marked with the same letter were not significantly different, according to the ANOVA test followed by the post hoc Tukey’s test for multiple comparison (*p* < 0.05).

**Figure 3:**
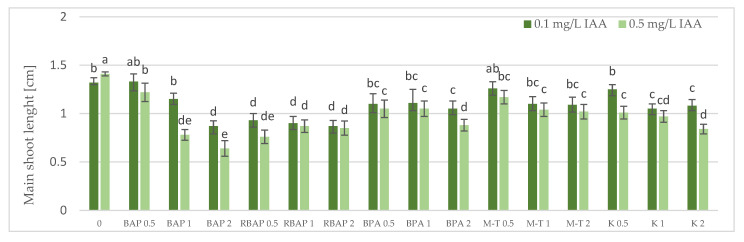
Effect of cytokinin type (BAP, RBAP, BPA, M-T and K) and concentration (0.5, 1 and 2 mg/L) on the length of *S. bulleyana* main shoot. Control (0)—shoots cultivated on MS medium only with auxin (IAA—0.1 mg/L and 0.5 mg/L). The values represent the mean ± standard error of three independent experiments. Means marked with the same letter were not significantly different, according to the ANOVA test followed by the post hoc Tukey’s test for multiple comparison (*p* < 0.05).

**Figure 4:**
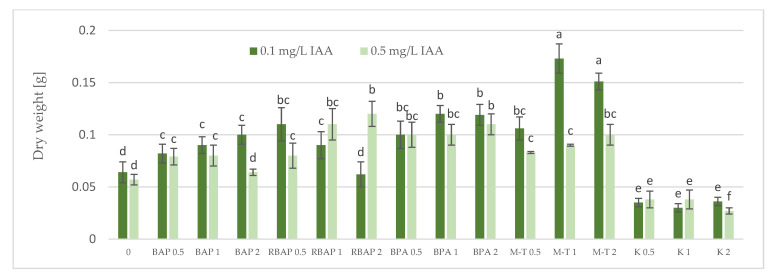
Effect of cytokinin type (BAP, RBAP, BPA, M-T and K) and concentration (0.5, 1 and 2 mg/L) on dry weight of *S. bulleyana* culture. Control (0)—shoots cultivated on MS medium only with auxin (IAA—0.1 mg/L and 0.5 mg/L). The values represent the mean ± standard error of three independent experiments. Means marked with the same letter were not significantly different, according to the ANOVA test followed by the post hoc Tukey’s test for multiple comparison *(p* < 0.05).

Auxin at a concentration of 0.1 mg/L turned out to be, in most cases, much more favorable for the growth of *S. bulleyana* cultures, judged by the proliferation coefficient, frequency of response, increase in dry matter and, often, the length of the main shoot (Figure 1, Figure 2, Figure 3 and Figure 4). In contrast, a higher IAA concentration clearly limited the effect of the cytokinins on shoot proliferation.

Micropropagation of various *Salvia* species using different natural and synthetic cytokinins, including purine and urea derivatives, has been earlier reported [14,15,22,23,24,25,26]. The growth regulators with a purine skeleton in their molecule were selected for this study because they have been documented to encourage multiplication in *Salvia* spp. [22,23,24]. However, most of the previous studies used traditional cytokinins such as BAP and K [14,15,16,25,26]. BAP displayed strong proliferative potential for *S. canadensis* [25], with a mean value of 25 new shoots on nodal explants being obtained within 45 days, but the proliferation coefficients for shoot tips and nodal explants of different sage species obtained in the presence of BAP were not usually high and only rarely exceeded three new shoots [23,24,26,27]. The authors associate this with the strong tendency for apical dominance noticed in plants of this genus, which can result in the slow growth of regenerated shoots [15,26]. Therefore, in addition to two traditional cytokinins, BAP and K, three promising BAP derivatives (RBAP, BPA, M-T) were selected for the present experiment, based on recent advanced achievements in the field [27,28,29]. 

All cytokinins, except 0.5 mg/L of K, significantly enhanced the regeneration of *S. bulleyana* shoots at almost all concentrations, manifested by a high percentage of response and proliferation coefficients (Figure 1 and Figure 2). The results revealed that all the advanced cytokinins tested (BPA, M-T and RBAP) achieved a significantly higher percentage of shoot regeneration than the classical phytohormones, BAP and K.

The highest proliferation coefficient was almost seven yielded shoots grown on an MS medium supplemented with 0.1 mg/L auxin and 2 mg/L BPA (Figure 2); in this case, the mean length of the main shoot exceeded 1 cm (Figure 3). The culture grown in the presence of BPA was also characterized by a high value of dry weight (Figure 4).

Lowering the BPA concentration in the medium to 1 mg/L, without changing the auxin concentration, resulted in a slight decrease in the multiplication ratio—up to 6.2; however, reducing BPA to 0.5 mg/L resulted in more than a twofold (3.2) decrease (Figure 2). Reducing this cytokinin level also gradually decreased the percentage of explants, giving a proliferative response. However, this factor remained relatively high, even at the lowest concentration used (90%) (Figure 1). 

BPA, although at a lower concentration (0.5 mg/L), is also proven to be optimal for stimulating bud regeneration in *S. viridis* cultures [27]. Moreover, BPA at a concentration of 2 mg/L has been found to increase the multiplication of *Dracecephalum forrestii* shoots [28].

A high multiplication coefficient (5.1–5.3) was also obtained for *S. bulleyana* in the presence of 1–2 mg/L RBAP and 0.1 mg/L IAA (Figure 2). In this case, 90–100% of the explants regenerated shoots/buds. However, despite being advantageous for proliferation, such concentrations of RBAP reduced shoot length, leaf size, and biomass accumulation (Figure 3 and Figure 4). Such findings are similar to those obtained for *S. viridis* cultures, where RBAP at all concentrations inhibited shoot elongation and increases in dry weight, giving results comparable to a control treatment (a medium only with auxin) [27]. On the other hand, in the present study, a lower RBAP concentration (0.5 mg/L) stimulated growth of the *S. bulleyana* culture with a slight increase in biomass (Figure 4). In that case, however, the multiplication coefficient drastically decreased (Figure 2). 

The other BAP derivative used in the experiment was the hydroxyl derivative, M-T (Figure 3). The concentration of M-T at 1 and 2 mg/L, in combination with 0.1 mg/L IAA, gave a 96–100% regeneration rate (Figure 1) and a relatively high multiplication coefficient (Figure 2). Although these were not optimal conditions for *S. bulleyana* proliferation, this combination of growth regulators gave the highest increase in dry weight of the culture (over 30-fold, i.e., 0.15–0.17 g within five weeks) (Figure 4). Also, in studies on other plant species, M-T demonstrated a positive effect on growth and shoot quality. This cytokinin was found to increase the multiplication factor of various plant in vitro cultures, stimulate their growth and the transformation of buds into shoots, increase shoot survival and support their rooting [6,7,29]. Moreover, M-T was shown to be the most effective cytokinin for biomass growth in cultures of *Prunus* [30], *Aloe* [31] and *Salvia* species [27].

In contrast to BAP derivatives, BAP and K (the furfuryl aminopurine derivative), especially in the lowest concentration used (0.5 mg/L), were not very effective in the propagation of *S. bulleyana* shoots. The shoot formation frequency on explants and the proliferation ratio recorded for 0.5 mg/L K in the medium were comparable to those obtained for the control medium, i.e., with auxin alone (Figure 2). Both cytokinins at higher concentrations (1–2 mg/L) inhibited shoot elongation, and the biomass of cultures grown on media with K was also lower than the shoots developed on the medium without cytokinins (Figure 4). The low dry weight of the culture on K-containing media was due to poor multiplication but, above all, to the poor growth of the culture: the formation of only small number of small leaves. Previously, K turned out to also be unfavorable for the shoot regeneration of *Salvia guaranitica* and *S. fruticosa* [15,24]. However, this is not the rule in the case of all sage species: *S. valentina* and *S. blancoana* demonstrated twice the proliferation ratio in the presence of K compared with BAP [26]. 

Summarizing, BAP derivatives—both hydroxylated at the 3-position of the benzyl ring and with a substituted N9 position in the purine ring—demonstrated increased activity in stimulating growth compared to BAP itself, which may be a result of the prolongated metabolism of these compounds in the plant [32].

### 3.2. Effect of Phytohormones on Metabolite Accumulation

**Figure 5:**
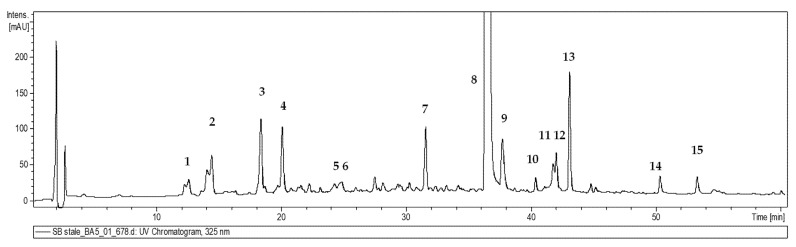
Representative UV chromatogram of the hydromethanolic extract of *S. bulleyana* shoot culture recorded by the UPLC- DAD/ESI-MS/MS system at 325 nm. Peak numbers correspond to the compound numbers in Table 1.

The phenolic profile of the obtained plant cultures was investigated using UPLC-DAD/ESI-MS/MS. Fifteen phenolic acids were identified in all extracts of the *S. bulleyana* shoots according to their retention time, UV spectra and mass spectra [12,19,20] (Table 1, Figure 5). All compounds were previously identified in other plants of the Salvia genus [12,19,20]. However, the level of the polyphenolic compounds was significantly influenced by the concentration of auxin and the type and concentration of cytokinin in the culture medium. Detailed contents of the individual compounds in shoots for individual treatments are presented in Table 2 and Table 3.

The predominant metabolite of hydromethanolic extract from the *S. bulleyana* culture was rosmarinic acid (RA). Its content in shoots grown on media with 0.1 mg/L IAA ranged from 8.24 to 33.67 mg/g dw (Table 2). The maximum level was found in an extract of shoots growing on the medium containing 0.1 mg/L IAA and 2 mg/L RBAP (Table 2). This amount of RA was twice as high as than that seen on the control medium, i.e., MS supplemented only with auxin (Table 2), and 4.5 times higher than on an MS medium without growth regulators (7.36 mg/g dw). The RA content gradually decreased when the RBAP concentration in the medium fell, reaching a level of 25.8 mg/g dw at 0.5 mg/L RBAP.

The addition of BPA and M-T to a medium supplemented with 0.1 mg/L IAA also improved RA biosynthesis in proliferating shoots. For example, RA accumulation in shoots cultivated on a medium containing 0.1 mg/L IAA and 2 mg/L BPA was approximately 29.3 mg/g dw, while with 0.5 mg/L, M-T was 27.2 mg/g dw (Table 2). However, these levels were about 15–20% less than in the shoots grown in optimal conditions, i.e., in the presence of 2 mg/L RBAP. 

In case of *S. viridis*, supplementation of the MS medium with 2 mg/L BPA resulted in the highest content of RA in the shoots [27]. The RA level achieved under these conditions was twice as high as in the control treatment (MS medium only with auxin). BPA was also the most effective for RA biosynthesis in *D. forrestii* shoots [28]. However, in the latter study, the higher concentration of this cytokinin was optimal (5 mg/L), resulting in a five-fold increase in RA levels compared to the treatment without cytokinin in the medium.

Meanwhile, M-T particularly intensely stimulated phenylpropanoid biosynthesis in *S. viridis* shoots grown in vitro. The level of the main compound from this group, i.e., verbascoside, increased two- to five-fold, depending on M-T concentration and compared to the treatment on MS with 0.1 mg/L IAA [27]. 

Supplementing the growth medium with BAP and K was not beneficial for RA accumulation in *S. bulleyana* shoots. At the lower concentrations, BAP and K (0.5 and 1 mg/L) in the presence of 0.1 mg/L IAA, RA content in the culture was lower than in the control treatment (Table 2). Similar results were obtained previously for *S. viridis* culture and in the *Scutellaria alpina* shoots, where BAP itself did not stimulate verbascoside production [27,33]. In contrast, the accumulation of most hydroxybenzoic and hydroxycinnamic acid derivatives in *Merwilla plumbe* plantlets was significantly enhanced in the presence of BAP, but only to a slight degree by M-T [34]. However, it is difficult to define the rule regarding the influence of different BAP derivatives on the production of polyphenolic compounds because existing studies are too scarce.

According to the present investigation, RA production was less efficient in shoots of *S. bulleyana* grown at higher IAA concentrations (Table 3), and smaller differences in RA levels were found between shoots grown at different concentrations of different cytokinins. However, RBAP, BPA and M-T were still more favorable for RA production, with RA levels ranging from 15 to 20.7 mg/g dw following the treatment with different concentrations of these three benzylaminopurine derivatives (Table 3).

**Figure 6:**
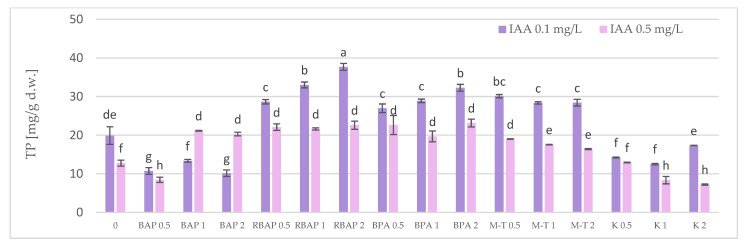
Effect of cytokinin type (BAP, RBAP, BPA, M-T and K) and concentration (0.5, 1 and 2 mg/L) on total phenolic (TP) content in shoot culture of *S. bulleyana*. Control (0)—shoots cultivated on MS medium only with auxin (IAA—0.1 mg/L and 0.5 mg/L). The values represent the mean *±* standard error of three independent experiments. Means marked with the same letter were not significantly different, according to the ANOVA test followed by the post hoc Tukey’s test for multiple comparison (*p* < 0.05).

A similar tendency was noted with respect to total phenolic (TP) content, due to the dominant RA share among polyphenols (Figure 6). The highest achieved TP level (37.7 mg/g dw) was found in shoots cultured on a medium with 0.1 mg/L IAA and 2 mg/L RBAP (Figure 6); this value was twice as high as that found in the control and three times that observed in the above-ground parts of two-year-old soil-grown plants [12].

The second-most prevalent compound in the *S. bulleyana* culture was salvianolic acid K (SAK). The addition of 0.1 mg/L auxin alone to the growth medium increased SAK accumulation 3.5 times compared to the MS medium without phytohormones, i.e., from 0.347 to 1.208 mg/g dw. However, adding cytokinins to the auxin-containing medium stimulated SAK biosynthesis only in the presence of 2 mg/L RBAP (1.37 mg/g dw). The remaining treatments, apart from 0.5 mg/L M-T and BPA, resulted in lower SAK levels than in the control medium, i.e., with auxin alone (Table 2). The effect of the higher IAA concentration (0.5 mg/L) was slightly weaker; it caused only a two-fold increase in the SAK content compared to the medium without any growth regulators (Table 3). A further increase in the SAK content was noted when the medium was supplemented with BAP at concentrations of 1 and 2 mg/L and its riboside (RBAP) at a concentration of 0.5 mg/L, but the obtained values were significantly lower than those described for optimal treatment (Table 2).

For most phenolic acids quantified in the study, shoots grown on a medium with 0.1 mg/L auxin were more productive and reached their highest amounts in combination with 2 mg/L RBAP (Table 2), followed by treatments with 1 mg/L RBAP, 2 mg/L BPA and 1 mg/L M-T. Exceptionally, higher auxin content (0.5 mg/L) in the medium yielded higher levels of protolithospermic acid isomers and salvianolic acid F isomers (Table 3).

In conclusion, substituted BAP derivatives, especially RBAP, significantly stimulated the biosynthesis of most polyphenolic compounds in *S. bulleyana* cultures, while BAP itself and K inhibited the polyphenol accumulation. 

### 3.3. Establishment of Optimal Conditions Based on the Obtained Results

Despite clear answers in relation to the individual analyzed parameters, the final decision regarding the optimal medium for high productivity of *S. bulleyana* cultivation was difficult. The highest multiplication factor was obtained for explants grown on an MS medium with the addition of 0.1 mg/L IAA and 2 mg/L BPA, the highest increase in biomass was in the culture grown on the medium supplemented with 0.1 mg/L IAA and 1 mg/L M-T and the highest polyphenol content was seen in the shoots on the medium with 0.1 mg/L IAA and 2 mg/L RBAP. Varied responses are commonly reported with respect to plant growth and compound production. Such an effect was observed in the case of *Colenonema pulchellum* shoots, whose maximum regeneration was observed in the presence of 13.6 M TDZ, while the shoots growing in the presence of 13.3 M BAP had a higher content of total phenols and flavonoids [35]. Similarly, the multiplication ratio and the biomass of *Knautia sarajevensis* culture grown in the medium containing 2.0 mg/L zeatin was significantly higher than those observed for other treatments, but the highest content of total phenols was recorded for shoots cultivated in media containing 1.0 mg/L kinetin, followed by 2.0 mg/L BAP [36]. 

Due to the lack of a clear conclusion regarding the optimal medium, the TOPSIS analysis was included in the present study. TOPSIS is an analytical multi-criteria decision-making technique that allows the selection of a preferred alternative that is closest to the positive ideal solution and furthest from the negative ideal solution [37]. This method has been used with a positive effect when selecting the media with different sucrose and vitamin content for the hairy roots of *S. bulleyana* [21]. It can be especially helpful when comparing multiple variants when it is important to optimize several parameters.

The first step of this method is to select the parameters that need to be optimized. In the present study, four such factors were chosen: total phenolic acid content, RA level, dry biomass and proliferation ratio. The next step is to give weight to individual parameters. For the aim, which is the highest productivity of *S. bulleyana* shoot culture, we decided that the most important factors are the production of compounds (both all compounds and the dominant, biologically active RA) and the increase in total dry weight, because this parameter has a significant impact on the productivity. These factors received weights of 0.3. The multiplication factor received a lower weight of 0.1. Shoot proliferation ability is necessary to maintain the continuity of the culture, but not as important as the increase in biomass, which could result not only from the high multiplication factor but also from the growth of organs and tissues. This weighting of results is determined by the overriding study aim and would be estimated differently if that goal was to obtain as many new plants as possible, which is the case in horticulture. 

After the calculations, the highest performance score was found for shoots grown on media with 0.1 mg/L IAA and 2 mg/L BPA or with 0.1 mg/L IAA and 1 mg/L M-T (0.76), and, hence, these media might be regarded as the most effective for high phenolic acid productivity in *S. bulleyana* culture (Table 4).

Thus, the final selection has been reduced to the two most effective treatments. The culture obtained on the medium with 0.1 mg/L IAA and 1 mg/L M-T demonstrated the highest dry weight, with quite high RA and TP content and quite favorable proliferation coefficient; in contrast, the treatment on the medium with 0.1 mg/L IAA and 2 mg/L BPA was characterized by the highest proliferation potential with effective RA and total polyphenol production and reasonable biomass accumulation.

A parameter that should be also taken into account is the price of individual growth regulators; this is notthe total cost of cultivation but also a factor that could affect the profitability of the culture. M-T is twice as cheap as BPA, 118.50 € and 543.4 € per gram, respectively (according to the price list as of 12 November 2022, of Duchefa Biochemie, BH Haarlem, The Netherlands). In addition, in selected media, M-T is used in half the concentration (1 mg/L) of BPA (2 mg/L). Therefore, an MS medium supplemented with 0.1 mg/L IAA and 1 mg/L M-T was ultimately selected as optimal for further experiments.

## 4. Conclusions

The present study reported an optimized propagation and growth protocol for *S. bulleyana* using shoot tip explants. Optimization of the type, concentration and combination of phytohormones is essential not only for successful shoot growth and multiplication but also for high bioactive compound production. The present study used the technique for order of preference by similarity to the ideal solution (TOPSIS) tool to determine the optimum method based on the wide range of considered parameters and the ambiguity of the results. Based on our findings, the substituted BAP derivatives have been found particularly advantageous for the accumulation of biomass and bioactive compounds in *S. bulleyana* cultures. The most efficient for phenolic acid accumulation in shoots turned out to be the MS medium containing 0.1 mg/L IAA and 1 mg/L M-T. Under these conditions, a highly productive culture yielded a phenolic acid content exceeding 28.4 mg/g dry weight within five weeks, which was 2.5 times higher than in the above-ground parts of two-year-old mother plants. Therefore, this culture can potentially be used for the production of medicinally relevant polyphenols.

## Figures and Tables

**Table 1:** Polyphenols of *Salvia bulleyana* shoot culture extracts detected by UPLC- DAD/ESI-MS/MS.

	Rt	Tentative Compound	[M-H]^-^	Fragmentation Ion
1	12.6	Caffeoyl-threonic acid I (CTA I)	297	179, 161, 135
2	14.5	Caffeoyl-threonic acid II (CTA II)	297	179, 161, 135
3	18.6	Caffeic acid CA	179	135
4	20.1	Caffeoyl-threonic acid III (CTA III)	297	179, 161, 135
5	24.2	Protolithospermic acid isomer I (PLA I)	357	313, 269, 203
6	24.8	Protolithospermic acid isomer II (PLA II)	357	313, 269, 203
7	31.5	Rosmarinic acid hexoside (RAH)	521	359
8	36.5	Rosmarinic acid (RA)	359	197, 179, 161
9	37.5	Salvianolic acid K (SAK)	555	537,493, 359, 313, 269
10	40.2	Lithospermic acid isomer (LA)	537	493, 359
11	41.6	Dehydrorosmarinic acid (DRA)	343	325, 223, 197, 179, 135
12	42.2	Caffeic acid derivative (CAD)	343	181, 161
13	43.5	Methyl rosmarinate (MR)	373	179, 135
14	50.8	Salvianolic acid F isomer I (SAF I)	313	269, 161
15	53.6	Salvianolic acid F isomer II (SAF II)	313	269, 203, 161

**Table 2:** Phenolic compound content (mg/g dw) in shoot culture of *S. bulleyana* grown on MS medium with 0.1 mg/L IAA and different cytokinins at different concentrations.

Phytohormon combination	Compound [mg/g dw]													
	CTA I	CTA II	CA	CTA III	PSL I	PSL II	RAH	RA	SAK	LS	DRA+CAD	RM	SAF I	SAF II
0	tr	0.29 ± 0.036cd	0.28 ± 0.037ab	0.176 ± 0.021e	0.066 ± 0.008ef	0.062 ± 0.007cd	0.125 ± 0.018g	16.76 ± 1.86e	1.208 ± 0.16ab	0.307 ± 0.039b	0.144 ± 0.020d	0.146 ± 0.017gh	0.163 ± 0.021cd	0.106 ± 0.012d
BAP 0.5	tr	0.036 ± 0.003h	0.249 ± 0.017bc	0.039 ± 0.003h	0.214 ± 0.005b	0.108 ± 0.010b	0.023 ± 0.004i	8.87 ± 0.74h	0.481 ± 0.044h	tr	0.062 ± 0.006g	0.171 ± 0.022gf	0.283 ± 0.014b	0.190 ± 0.013b
BAP 1	tr	0.017 ± 0.001i	0.15 ± 0.001h	0.022 ± 0.001i	0.128 ± 0.004c	0.064 ± 0.001d	0.023 ± 0.001i	11.77 ± 0.30fg	0.654 ± 0.027f	tr	0.094 ± 0.004e	0.194 ± 0.008f	0.14 ± 0.001d	0.101 ± 0.001d
BAP 2	tr	0.05 ± 0.002g	0.284 ± 0.001a	0.044 ± 0.001h	0.285 ± 0.001a	0.210 ± 0.001a	tr	8.24 ± 0.76h	0.242 ± 0.020j	tr	0.064 ± 0.007g	0.143 ± 0.029gh	0.33 ± 0.008a	0.238 ± 0.005a
RBAP 0.5	0.148 ± 0.002a	0.485 ± 0.003a	0.186 ± 0.001f	0.371 ± 0.008a	tr	0.049 ± 0.003e	0.243 ± 0.007d	25.82 ± 0.51d	0.708 ± 0.025e	0.122 ± 0.001e	0.143 ± 0.003d	0.262 ± 0.007d	0.071 ± 0.001g	0.046 ± 0.001g
RBAP 1	0.122 ± 0.007b	0.418 ± 0.014b	0.233 ± 0.006cd	0.348 ± 0.029a	tr	0.067 ± 0.001cd	0.341 ± 0.032c	29.58 ± 0.27b	0.675 ± 0.191efg	0.479 ± 0.095a	0.23 ± 0.039b	0.288 ± 0.034cd	0.126 ± 0.013e	0.094 ± 0.012de
RBAP 2	0.064 ± 0.001e	0.304 ± 0.005c	0.226 ± 0.006d	0.254 ± 0.004b	tr	0.064 ± 0.001d	0.463 ± 0.014a	33.67 ± 0.80a	1.37 ± 0.037a	0.415 ± 0.019a	0.306 ± 0.008a	0.348 ± 0.006b	0.125 ± 0.001e	0.095 ± 0.001e
BPA 0.5	0.077 ± 0.004d	0.277 ± 0.014d	0.187 ± 0.008f	0.204 ± 0.006d	0.028 ± 0.005g	0.061 ± 0.006de	0.187 ± 0.003f	24.13 ± 0.96d	0.962 ± 0.039b	0.205 ± 0.033d	0.183 ± 0.013c	0.299 ± 0.012c	0.090 ± 0.004f	0.072 ± 0.006f
BPA 1	0.096 ± 0.002c	0.313 ± 0.005c	0.246 ± 0.004bc	0.222 ± 0.001c	0.131 ± 0.003c	0.102 ± 0.001b	0.199 ± 0.006e	25.80 ± 0.4d	0.801 ± 0.02d	0.281 ± 0.005c	0.209 ± 0.004b	0.224 ± 0.002e	0.151 ± 0.002c	0.117 ± 0.002c
BPA 2	0.064 ± 0.005e	0.218 ± 0.020e	0.234 ± 0.013cd	0.170 ± 0.009e	0.062 ± 0.007f	0.106 ± 0.004b	0.218 ± 0.013e	29.33 ± 0.73b	0.615 ± 0.016fg	0.339 ± 0.004b	0.229 ± 0.04b	0.404 ± 0.017a	0.165 ± 0.009c	0.124 ± 0.009c
M-T 0.5	0.073 ± 0.001d	0.276 ± 0.002d	0.18 ± 0.006f	0.2 ± 0.002d	tr	0.054 ± 0.001e	0.24 ± 0.003d	27.19 ± 0.37bc	1.02 ± 0.02b	0.203 ± 0.006d	0.15 ± 0.002d	0.396 ± 0.008a	0.074 ± 0.001g	0.05 ± 0.003g
M-T 1	0.132 ± 0.005b	0.423 ± 0.014b	0.209 ± 0.003e	0.348 ± 0.006a	0.061 ± 0.001f	0.093 ± 0.005b	0.423 ± 0.002b	25.38 ± 0.25d	0.615 ± 0.008fg	0.124 ± 0.002e	0.1 ± 0.003e	0.283 ± 0.003c	0.105 ± 0.001f	0.059 ± 0.001g
M-T 2	0.066 ± 0.002e	0.236 ± 0.011e	0.186 ± 0.005f	0.182 ± 0.008e	0.019 ± 0.002g	0.071 ± 0.001c	0.195 ± 0.009ef	26.03 ± 0.78cd	0.561 ± 0.011g	0.138 ± 0.008e	0.182 ± 0.004c	0.389 ± 0.009a	0.108 ± 0.005f	0.069 ± 0.003f
K 0.5	0.025 ± 0.001f	0.087 ± 0.002f	0.156 ± 0.004h	0.108 ± 0.001f	0.070 ± 0.002e	0.047 ± 0.002e	0.042 ± 0.001h	12.41 ± 0.1f	0.745 ± 0.002e	0.033 ± 0.001g	0.103 ± 0.001e	0.168 ± 0.002g	0.147 ± 0.004cd	0.093 ± 0.001e
K 1	0.012 ± 0.001g	0.052 ± 0.002g	0.166 ± 0.004g	0.082 ± 0.003g	0.099 ± 0.007d	0.066 ± 0.002cd	0.025 ± 0.003i	10.71 ± 0.14g	0.803 ± 0.007d	0.039 ± 0.001g	0.079 ± 0.005f	0.134 ± 0.001h	0.15 ± 0.005c	0.107 ± 0.003d
K 2	0.014 ± 0.001g	0.055 ± 0.001g	0.166 ± 0.001g	0.084 ± 0.001g	0.071 ± 0.002e	0.06 ± 0.001d	0.041 ± 0.001h	15.31 ± 0.03e	0.868 ± 0.001c	0.051 ± 0.001f	0.15 ± 0.001d	0.255 ± 0.004d	0.146 ± 0.001cd	0.092 ± 0.002e

The values represent the mean ± standard error of three independent experiments. Means marked with the same letter for the same metabolite were not significantly different, according to the ANOVA test followed by the post hoc Tukey’s test for multiple comparison (*p* < 0.05); tr-traces.

**Table 3:** Phenolic compound content (mg/g dw) in shoot culture of *S. bulleyana* grown on MS medium with 0.5 mg/L IAA and different cytokinins at different concentrations.

Phytohormon combination	Compound [mg/g dw]													
	CTA I	CTA II	CA	CTA III	PSL I	PSL II	RAH	RA	SAK	LS	DRA+CAD	RM	SAF I	SAF II
0	0.045 ± 0.003e	0.186 ± 0.013e	0.179 ± 0.014fg	0.113 ± 0.007g	0.042 ± 0.003h	0.040 ± 0.002i	0.080 ± 0.007b	10.76 ± 0.62c	0.774 ± 0.06c	0.197 ± 0.014a	0.092 ± 0.008c	0.094 ± 0.006h	0.104 ± 0.008i	0.068 ± 0.004i
BAP 0.5	tr	0.024 ± 0.002h	0.255 ± 0.015d	0.030 ± 0.002i	0.272 ± 0.006ab	0.15 ± 0.012bc	tr	6.64 ± 0.55d	0.367 ± 0.032f	tr	0.039 ± 0.002f	0.081 ± 0.009h	0.345 ± 0.027de	0.239 ± 0.022f
BAP 1	tr	tr	0.152 ± 0.001g	0.040 ± 0.001i	0.119 ± 0.001e	0.126 ± 0.002d	0.030 ± 0.001d	19.00 ± 0.09a	0.899 ± 0.005b	tr	0.165 ± 0.001b	0.382 ± 0.002a	0.130 ± 0.002h	0.108 ± 0.001h
BAP 2	tr	0.033 ± 0.001g	0.197 ± 0.005f	0.075 ± 0.002h	0.083 ± 0.003g	0.110 ± 0.005e	0.044 ± 0.001c	17.83 ± 0.39a	0.979 ± 0.017a	0.055 ± 0.002d	0.198 ± 0.005a	0.325 ± 0.01b	0.210 ± 0.009g	0.57 ± 0.009a
RBAP 0.5	0.103 ± 0.001b	0.327 ± 0.001b	0.572 ± 0.019a	0.205 ± 0.013c	0.278 ± 0.011a	0.185 ± 0.025b	0.161 ± 0.001a	18.02 ± 0.44a	0.809 ± 0.158abc	0.07 ± 0.003c	0.173 ± 0.019ab	0.206 ± 0.024ef	0.614 ± 0.069a	0.343 ± 0.057cd
RBAP 1	0.126 ± 0.011ab	0.372 ± 0.034ab	0.536 ± 0.009a	0.295 ± 0.023ab	0.287 ± 0.005a	0.135 ± 0.006d	0.076 ± 0.001b	18.70 ± 0.08a	0.196 ± 0.01h	tr	0.092 ± 0.007c	0.233 ± 0.019de	0.401 ± 0.025c	0.18 ± 0.026fg
RBAP 2	0.058 ± 0.003d	0.189 ± 0.011e	0.581 ± 0.029a	0.142 ± 0.01f	0.265 ± 0.026ab	0.283 ± 0.015a	0.049 ± 0.004c	18.84 ± 0.87a	0.568 ± 0.016e	0.085 ± 0.004b	0.188 ± 0.012a	0.252 ± 0.018de	0.676 ± 0.026a	0.429 ± 0.021b
BPA 0.5	0.14 ± 0.024a	0.457 ± 0.054a	0.395 ± 0.034b	0.314 ± 0.015a	0.293 ± 0.035ab	0.176 ± 0.015b	0.076 ± 0.001b	19.61 ± 2.18ab	0.166 ± 0.005i	0.039 ± 0.016d	0.027 ± 0.008g	0.148 ± 0.033g	0.488 ± 0.03b	0.333 ± 0.023c
BPA 1	0.142 ± 0.012a	0.436 ± 0.037a	0.323 ± 0.032bc	0.261 ± 0.018b	0.277 ± 0.026ab	0.142 ± 0.014bcd	0.029 ± 0.002d	17.07 ± 1.17ab	0.114 ± 0.013j	tr	0.055 ± 0.009e	0.143 ± 0.012g	0.409 ± 0.036c	0.276 ± 0.022de
BPA 2	0.079 ± 0.003c	0.29 ± 0.011c	0.294 ± 0.009c	0.205 ± 0.008c	0.166 ± 0.007d	0.125 ± 0.007d	0.056 ± 0.002c	20.74 ± 0.88a	0.374 ± 0.019f	0.058 ± 0.011d	0.085 ± 0.024cd	0.18 ± 0.014f	0.305 ± 0.008e	0.201 ± 0.007f
M-T 0.5	0.065 ± 0.001d	0.221 ± 0.003d	0.306 ± 0.001c	0.162 ± 0.001e	0.097 ± 0.005f	0.102 ± 0.001e	0.04 ± 0.001c	16.71 ± 0.05ab	0.26 ± 0.002g	0.02 ± 0.001e	0.072 ± 0.001d	0.285 ± 0.001c	0.382 ± 0.002c	0.282 ± 0.003d
M-T 1	0.085 ± 0.003c	0.279 ± 0.007c	0.206 ± 0.001e	0.215 ± 0.001c	0.043 ± 0.008h	0.076 ± 0.001g	0.05 ± 0.001c	15.86 ± 0.04b	0.204 ± 0.004h	tr	0.024 ± 0.001g	0.212 ± 0.01e	0.191 ± 0.006g	0.11 ± 0.001h
M-T 2	0.068 ± 0.002d	0.215 ± 0.008d	0.229 ± 0.003d	0.178 ± 0.005d	0.098 ± 0.001f	0.062 ± 0.001h	0.033 ± 0.002d	14.59 ± 0.11b	0.243 ± 0.004g	tr	0.047 ± 0.002f	0.255 ± 0.003d	0.233 ± 0.003f	0.146 ± 0.001g
K 0.5	0.015 ± 0.001f	0.063 ± 0.002f	0.213 ± 0.004e	0.074 ± 0.001h	0.115 ± 0.005e	0.09 ± 0.001f	0.033 ± 0.001d	11.07 ± 0.05c	0.644 ± 0.004d	0.044 ± 0.004d	0.074 ± 0.001d	0.171 ± 0.007f	0.187 ± 0.012g	0.141 ± 0.005g
K 1	tr	tr	0.285 ± 0.009c	tr	0.209 ± 0.002c	0.157 ± 0.002c	tr	6.15 ± 0.80d	0.701 ± 0.098d	tr	0.056 ± 0.011e	0.127 ± 0.009g	0.356 ± 0.001d	0.296 ± 0.002c
K 2	tr	tr	0.289 ± 0.005c	tr	0.255 ± 0.001b	0.153 ± 0.001c	tr	5.14 ± 0.12d	0.53 ± 0.011e	tr	0.078 ± 0.005cd	0.101 ± 0.007h	0.365 ± 0.001d	0.307 ± 0.002c

The values represent the mean *±* standard error of three independent experiments. Means marked with the same letter for the same metabolite were not significantly different, according to the ANOVA test followed by the post hoc Tukey’s test for multiple comparison (*p* < 0.05); tr-traces.

**Table 4:** Calculation of performance score for individual treatments using TOPSIS.

Performance Score	IAA 0.1 g/L	IAA 0.5 mg/L
0	0.346	0.162
BAP 0.5	0.239	0.205
BAP 1	0.308	0.421
BAP 2	0.295	0.365
RBAP 0.5	0.637	0.425
RBAP 1	0.673	0.509
RBAP 2	0.641	0.548
BPA 0.5	0.589	0.487
BPA 1	0.700	0.436
BPA 2	0.764	0.542
M-T 0.5	0.663	0.380
M-T 1	0.763	0.386
M-T 2	0.758	0.386
K 0.5	0.186	0.160
K 1	0.100	0.056
K 2	0.195	0.065

## Data Availability

Data are contained within the article.
